# Anti-inflammatory effect of afatinib (an EGFR-TKI) on OGD-induced neuroinflammation

**DOI:** 10.1038/s41598-019-38676-7

**Published:** 2019-02-21

**Authors:** Yen-Ju Chen, Chia-Chi Hsu, Young-Ji Shiao, Hsiang-Tsui Wang, Yu-Li Lo, A. M. Y. Lin

**Affiliations:** 1 Institute of Pharmacology, National Yang-Ming University, Taipei, Taiwan; 2 Department of Oncology, National Taiwan University Hospital, Taipei, Taiwan; 3 National Research Institute of Chinese Medicine, Ministry of Health and Welfare, Taipei, Taiwan; 4 Faculty of Pharmacy, National Yang-Ming University, Taipei, Taiwan; 5 Department of Medical Research, Taipei-Veterans General Hospital, Taipei, Taiwan

## Abstract

Activated epidermal growth factor receptor (EGFR) has been proposed in the pathophysiology of neurodegenerative diseases. In the present study, the anti-inflammatory effect of afatinib, an EGFR-tyrosine kinase inhibitor (EGFR-TKIs) was investigated using CTX-TNA2 cells and primary cultured astrocytes subjected to oxygen/glucose deprivation (OGD). We found that OGD induced EGFR phosphorylation and activated subsequent signaling pathways, including phosphorylation of AKT and extracellular signal-regulated kinases (ERK). Afatinib blocked OGD-induced phosphorylation of EGFR, AKT and ERK. At the same time, afatinib attenuated OGD-induced elevations in glial fibrillary acidic protein (a biomarker of activated astrocytes) and proliferating cell nuclear antigen expression (a cell proliferating biomarker) as well as hypoxia-induced migratory ability. Furthermore, afatinib decreased OGD-induced increases in cyclooxygenase-II and inducible nitric oxide synthase expression of the treated astrocytes as well as NO content in the culture medium. Moreover, afatinib attenuated OGD-induced caspase 1 activation (a biomarker of inflammasome activation) and interleukin-1β levels (a pro-inflammatory cytokine). Collectively, afatinib could block OGD-induced EGFR activation and its downstream signaling pathways in astrocytes. Moreover, afatinib attenuated OGD-induced astrocyte activation, proliferation and inflammasome activation. These data support the involvement of EGFR activation in neuroinflammation. Furthermore, EGFR-TKIs may be promising in inhibiting neuroinflammation in the CNS neurodegenerative diseases.

## Introduction

Epidermal growth factor receptor (EGFR), a 171-kDa transmembrane glycoprotein with tyrosine kinase activity^1,2^, is expressed in epithelial and mesenchymal-origin tissues, including lung, skin and gastrointestinal systems^3^. In the central nervous system (CNS), EGFR is differentially expressed in neurons and glia during development as well as in adults. In the developing CNS, EGFR expression is detected in both neuron and glia. The maximal expression of EGFR is detected in rat astrocytes at day 19 postnatal and decreases thereafter, while EGFR expression in neurons begins at day 11 postnatal and is maintained at comparable levels in adulthood^4^. The role of glial EGFR in developing brain is critical to cell proliferation, migration, maturation and survival. In the adult brain, EGFR is mainly detected in neurons and neural progenitor cells in the subventricular zone^5^. Moreover, EGFR may exert its trophic action on neuronal stem cells resulting in cell survival, proliferation and differentiation into a specific cell type^5^.

Activation of EGFR reportedly couples to tyrosine kinase-induced autophosphorylation which subsequently activates multiple cellular signaling cascades. For example, EGFR activation activates PI3K-AKT and Raf-MAPK-ERK1/2 pathways^2,6,7^ to generate intracellular mediators which translocate into the nucleus to regulate DNA synthesis for cell growth and proliferation as well as to modulate cell survival, migration, differentiation and death^2,7^. The physiological role of EGFR has been delineated by mice lacking EGFR which showed systemic defects, including neurodegeneration and death^8^. Neuronal survival has been reported to directly depend on EGFR in neurons as well as indirect actions of EGFR in astrocytes^9^. Moreover, a neurotrophic role of EGFR in astrocytes has been suggested because significant EGFR expression reportedly regulates cytoskeleton and expression of glutamate transporter in cultured astrocytes^10^.

Pathologically, EGFR has been proposed to be involved in several neurodegenerative diseases, including Alzheimer’s disease, spinal cord injury and brain ischemia^11–14^. EGFR is scarcely detected in quiescent astrocytes in normal adult brain; however, EGFR reappears in reactive astrocytes in response to insults^15^. The EGFR re-activation is reportedly neuroprotective by inhibiting glutamate-induced neurotoxicity^15^ and guiding the migration of injured optic nerves^11^. In contrast, EGFR may also contribute to neurotoxicity since EGFR has been demonstrated to mediate oligomeric Aβ42-induced neurotoxicity in the Alzheimer’s animal models^14^. In the present study, the role of EGFR in neuroinflammation was investigated using oxygen/glucose deprivation (OGD), a well known *in vitro* model of brain ischemia. Furthermore, the anti-inflammatory effect of afatinib, a second-generation EGFR-tyrosine kinase inhibitor (EGFR-TKI), on OGD-induced neuroinflammation was studied *in vitro*. Clinically, afatinib is used for treating patients with non-small cell lung cancer^16,17^. The pharmacological mechanism of afatinib is to inhibit EGFR tyrosine kinase activity by covalently binding to the ATP-binding site in the kinase domains of EGFR^18^. Due to its ability to penetrate blood brain barrier (BBB)^19^, afatinib may be useful in inhibiting neuroinflammation in the CNS neurodegenerative diseases.

## Results

### Afatinib was not cytotoxic to CTX-TNA2 cells and primary cultured astrocytes

**Figure 1:**
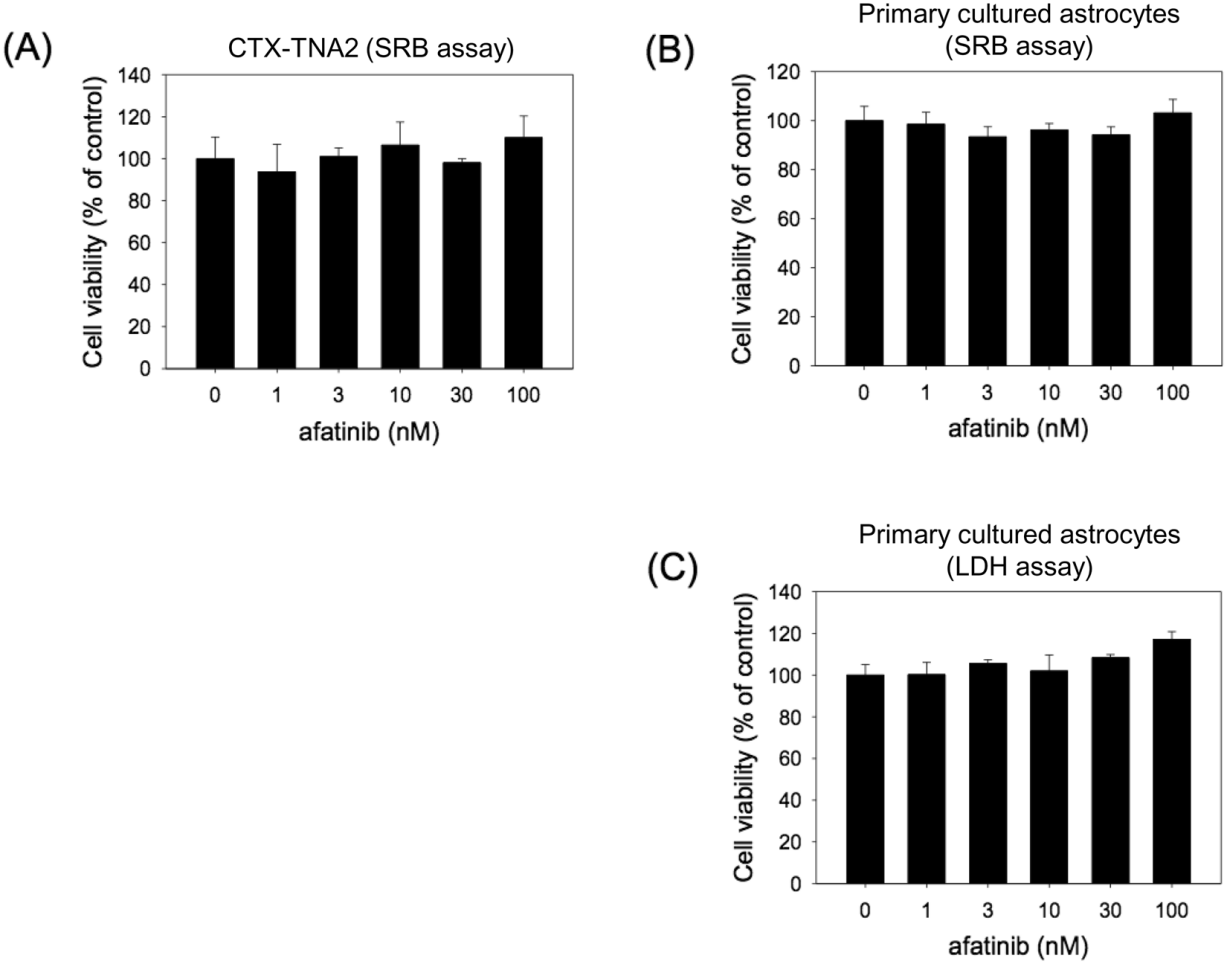
The effect of afatinib on the cell survial of CTX-TNA2 cells and primary cultured astrocytes. CTX-TNA2 cells and primary cultured astrocytes were treated with afatinib (1–100 nM) for 24 h. (**A**,**B**) Cell viability of CTX-TNA2 cells and primary cultured astrocytes was determined using SRB assay. (**C**) Cell viability of primary cultured astrocytes was determined by LDH assay. Values are normalized to the no drug condition and are the mean ± S.E.M. (n = 3/group).

The effect of afatinib on cell survival of CTX-TNA2 cells and primary cultured astrocytes was investigated using sulforhodamine B (SRB) assay. Both cells were incubated with afatinib (1–100 nM) for 24 h. Under normixic condition, afatinib did not affect the cell survival of CTX-TNA2 cells and primary cultured astrocytes while compared with the vehicle control (Fig. 1A,B). The lactate dehydrogenase (LDH) assay was employed to further confirm the effect of afatinib on primary cultured astrocytes. Incubation with afatinib (1–100 nM) did not induce significant increases in LDH levels (a hallmark of cell death) in the culture medium containing primary cultured astrocytes (Fig. 1C). These data indicate that afatinib was not cytotoxic to CTX-TNA2 cells and primary cultured astrocytes.

### Afatinib prevented OGD-induced EGFR phosphorylation and astrocyte activation

**Figure 2:**
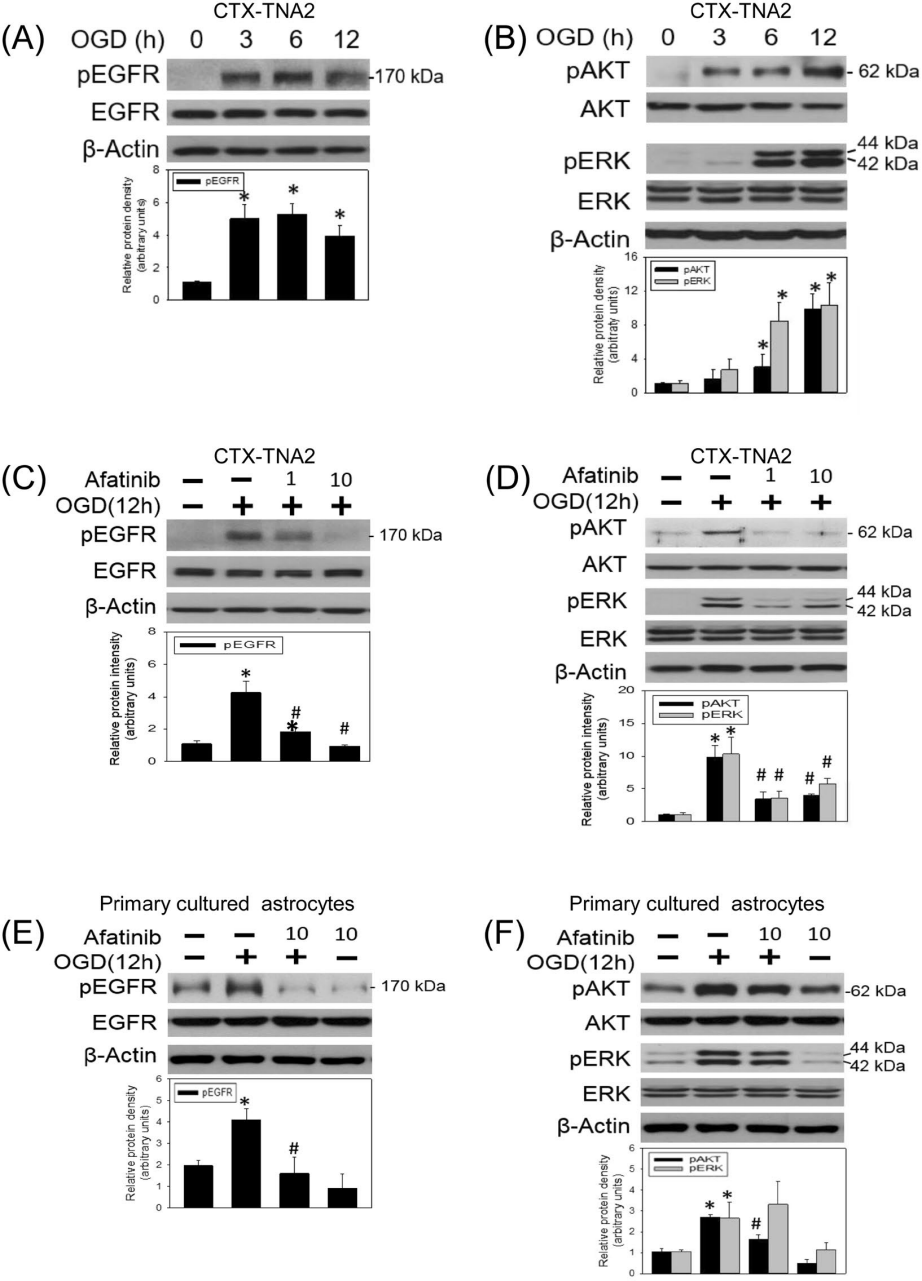
Afatinib blocked OGD-induced EGFR activation and downstream signalings. (**A**,**B**) CTX-TNA2 cells were exposed to oxygen-glucose deprivation (OGD) for various durations (3, 6, 12 h). Western blot assay was employed to measure (**A**) the levels of phospho-EGFR (pEGFR) and total-EGFR (EGFR) as well as (**B**) phospho-ERK (pERK), total-ERK (ERK), phospho-AKT (pAKT), total-AKT (AKT) and β-Actin. Each lane contained 40 μg protein for all experiments. Graphs show statistical results of pEGFR and EGFR (**A**) as well as pERK, ERK, pAKT and AKT (**B**) from relative optical density of bands on the blots. *P < 0.05 in the OGD group compared with the control by t-test. (**C**,**D**) CTX-TNA2 cells were exposed to OGD for 12 h. Afatinib (1, 10 nM) was included in the culture medium concomitantly with OGD exposure. (**E**,**F**) Primary cultured astrocytes were exposed to OGD for 12 h. Afatinib (10 nM) was included in the culture medium concomitantly with OGD exposure. Graphs show statistical results of pEGFR and EGFR (**C**,**E**) as well as pERK, ERK, pAKT and AKT (**D**,**F**) from relative optical density of bands on the blots. Values are the mean ± S.E.M. (n = 3/group). *P < 0.05 in the OGD group compared with the control, ^#^P < 0.05 in OGD plus afatinib compared with OGD alone by Kruskal-Wallis test and followed by Mann-Whitney U test as post-hoc method.

To study the anti-inflammatory effect of afatinib on brain ischemia, the involvement of EGFR in OGD was studied in CTX-TNA2 cells by exposing to OGD for varying durations (3, 6, 12 h). Western blot assay showed that OGD significantly induced EGFR phosphorylation after 3-h OGD and maintained phosphorylation of EGFR until at least 12-h OGD (Fig. 2A). Furthermore, OGD activated EGFR downstream signalings, including AKT and ERK phosphorylation in a time-dependent manner (Fig. 2B). The effect of afatinib on OGD-induced EGFR phosphorylation was investigated by treating afatinib concomitantly with OGD exposure. We found that afatinib (1 and 10 nM) attenuated OGD (12 h)-induced phosphorylation of EGFR (Fig. 2C) as well as the phosphorylation of AKT and ERK in the treated CTX-TNA2 cells (Fig. 2D). To confirm afatinib-induced inhibition of OGD-induced EGFR activation, primary cultured astrocytes were subjected to 12-h OGD with the presence of afatinib. Consistently, afatinib significantly blocked OGD-induced EGFR activation (Fig. 2E) and AKT phosphorylation but not ERK phosphorylation in primary cultured astrocytes (Fig. 2F). These data indicate that OGD activated EGFR phosphorylation and subsequent cellular signaling pathways; afatinib is capable of attenuating OGD-induced EGFR activation in astrocytes.

**Figure 3:**
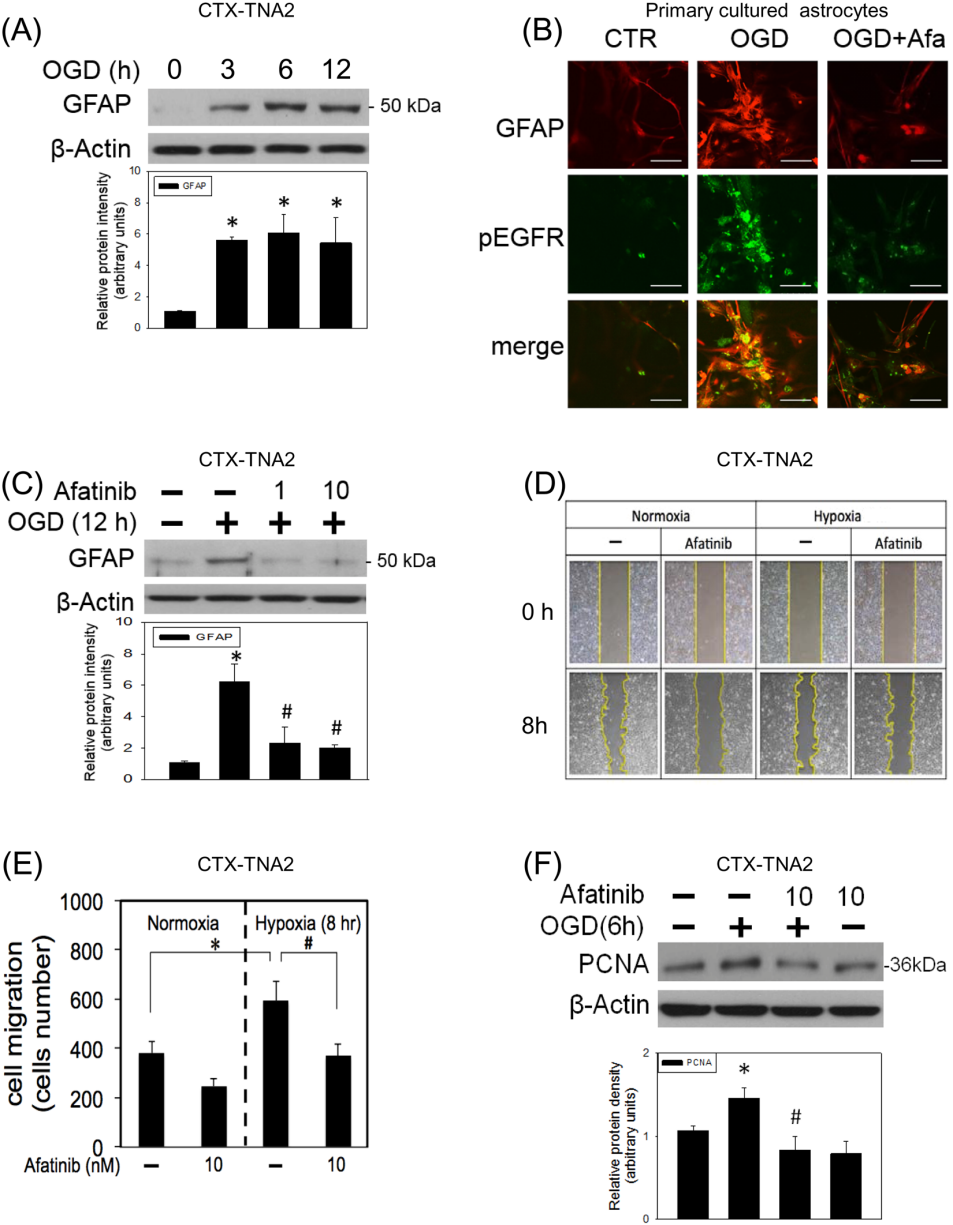
Afatinib prevented OGD-induced astrocyte activation. (**A**) CTX-TNA2 cells were exposed to oxygen-glucose deprivation (OGD) for various durations (3, 6, 12 h). Western blot assay was employed to measure GFAP. Each lane contained 40 μg protein for all experiments. Graphs show statistic results of GFAP from relative optical density of bands on the blots. Values are the mean ± S.E.M. (n = 3/group).*P < 0.05 in the OGD group compared with the control by t-test. (**B**) Representative confocal microscopic data showed co-localization of immunofluorescence of GFAP (Red) and pEGFR (Green). Primary cultured astrocytes were exposed to 12-h OGD. Afatinib (10 nM) was included in the culture medium concomitantly with OGD exposure. Scale bar: 20 μm. (**C**) CTX-TNA2 cells were exposed to 12-h OGD. Afatinib (1, 10 nM) was included in the culture medium concomitantly with OGD exposure. Graphs show statistic results of GFAP from relative optical density of bands on the blots. Values are the mean ± S.E.M. (n = 3/group). *P < 0.05 in the OGD group compared with the control, ^#^P < 0.05 in OGD plus afatinib compared with OGD alone by Kruskal-Wallis test and followed by Mann-Whitney U test as post-hoc method. (**D**) CTX-TNA2 cells were incubated with or without afatinib (10 nM) in normoxia and hypoxic (1% O_2_) chambers for 8 h. The cell migration of CTX-TNA2 cells was studied using a wound-healing assay. Representative microscopic data showed the cells at the identical location 8 h after the initiation of wound. (**E**) The statistical graph shows the migration quantified by counting the cells that had migrated in the cell-free area using Image J. Values are the mean ± S.E.M. (n = 3/group). (**F**) CTX-TNA2 cells were exposed to 6-h OGD. Afatinib was included in the culture medium concomitantly with OGD exposure. Graphs show statistic results of PCNA from relative optical density of bands on the blots. Values are the mean ± S.E.M. (n = 3/group). *P < 0.05 in the OGD group compared with the control, ^#^P < 0.05 in OGD plus afatinib compared with OGD alone by Kruskal-Wallis test and followed by Mann-Whitney U test as post-hoc method.

Neuroinflammation is thought to be central to CNS neurodegeneration^20^, and activated astrocytes is one of the sources for neuroinflammation^21^. In the present study, the effect of concomitant incubation of afatinib on OGD-induced astrocyte activation was investigated by measuring glial fibrillary acidic protein (GFAP, a hallmark of activated astrocytes) and astrocyte migration. OGD time-dependently increased GFAP in CTX-TNA2 cells. GFAP expression was increased after 3-h OGD and maintained elevated until 12-h OGD (Fig. 3A). At the same time, the immunofluorescent study demonstrated that intense immunoreactivity of phosphorylated GFAP colocalized with that of phosphorylated EGFR immunoreactivity in the OGD (12 h)-treated primary cultured astrocytes (Fig. 3B). Concomitant incubation of afatinib significantly attenuated OGD-induced elevation in colocalized immnofulorescent reactivities of EGFR and GFAP in the primary cultured astrocytes (Fig. 3B) and GFAP expression in CTX-TNA2 cells (Fig. 3C), respectively. Furthermore, afatinib attenuated hypoxia (1%)-induced cell migration in CTX-TNA2 cells (Figs. 3D,E). Moreover, afatinib reduced OGD-elevated proliferating cell nuclear antigen expression (PCNA, a biomarker of cell proliferation) (Fig. 3F). These data suggest that afatinib is capable of inhibiting OGD-induced astrocyte activation and proliferation.

### Afatinib inhibited OGD-induced inflammation

**Figure 4:**
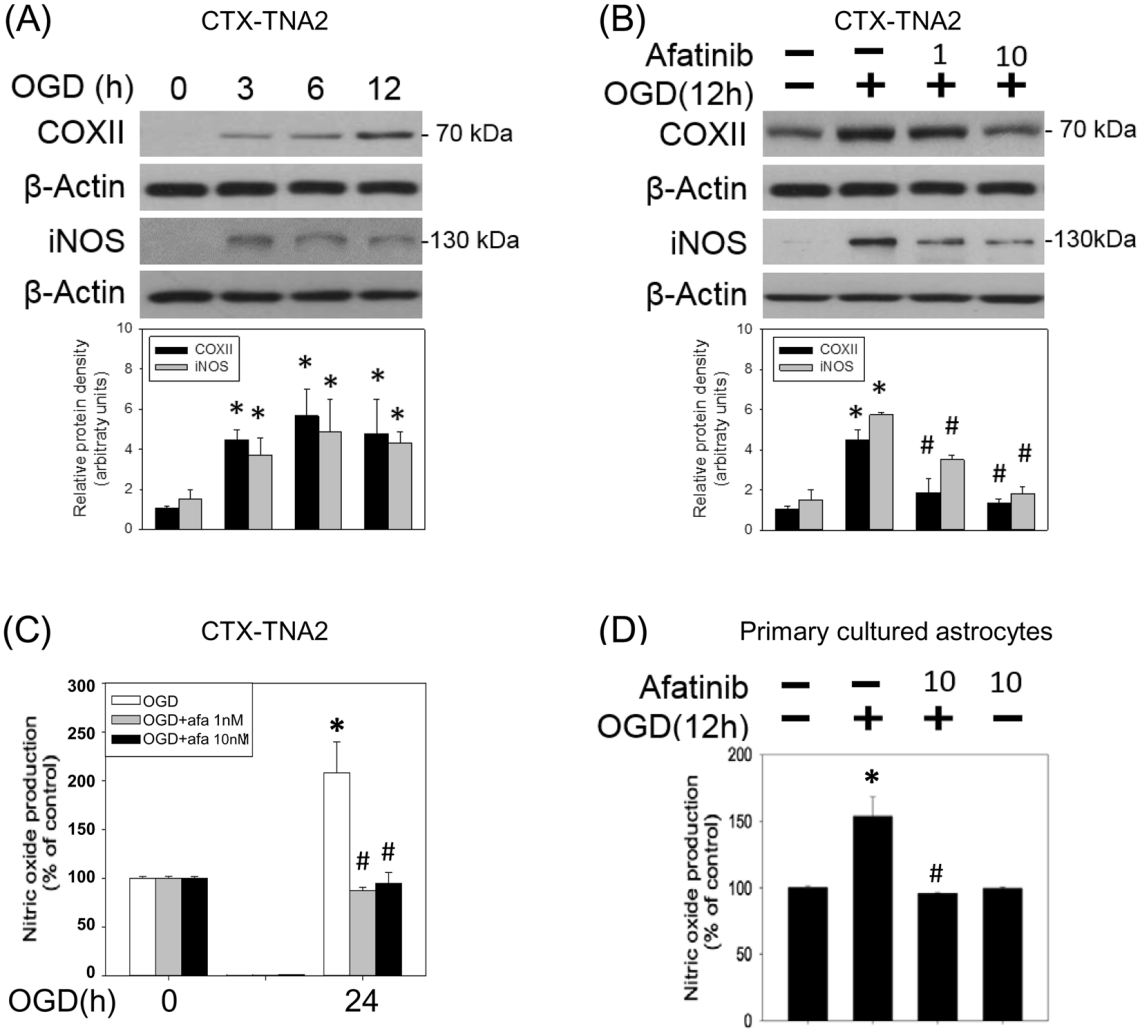
Afatinib attenuated OGD-induced increases in COX-II, iNOS and NO levels. (**A**) CTX-TNA2 cells were exposed to oxygen-glucose deprivation (OGD) for various durations (3, 6, 12 h). Western blot assay was employed to measure COX-II, iNOS and β-Actin. Each lane contained 40 μg protein for all experiments. Graphs show statistical results of COX-II and iNOS from relative optical density of bands on the blots. Values are the mean ± S.E.M. (n = 3/group).*P < 0.05 in the OGD group compared with the control by t-test. (**B**) CTX-TNA2 cells were exposed to 12-h OGD. Afatinib (1, 10 nM) was included in the culture medium concomitantly with OGD exposure. Graphs show statistical results of COX-II and iNOS levels from relative optical density of bands on the blots. Values are the mean ± S.E.M. (n = 3/group). (**C**) CTX-TNA2 cells and (**D**) Primary cultured astrocytes were exposed to 24 h-OGD or 12 h-OGD, respectively. Afatinib (1, 10 nM) was included in the culture medium concomitantly with OGD exposure. The levels of NO were measured using Griess reaction. Values are the mean ± S.E.M. (n = 3/group). *P < 0.05 in the OGD group compared with the control, ^#^P < 0.05 in OGD plus afatinib compared with OGD alone by Kruskal-Wallis test and followed by Mann-Whitney U test as post-hoc method.

To study the effect of afatinib on OGD-induced neuroinflammation, two proinflammatory enzymes, including inducible nitric oxide synthase (iNOS) and cyclo-oxygenase (COX)-II were measured. Western blot assay showed that OGD significantly increased iNOS and COX-II levels in the CTX-TNA2 cells (Fig. 4A). Concomitant incubation of afatinib inhibited OGD-induced elevation in iNOS and COX-II levels (Fig. 4B). Furthermore, the nitrite/nitrate (oxidized products of NO) in the culture medium containing CTX-TNA2 cells was determined using Griess reaction assay. Our data suggested that OGD (24 h) increased NO levels more than 2 folds compared with that in the control group; concomitant incubation of afatinib reduced OGD-induced NO accumulation to control levels (Fig. 4C). Similarly, afatinib inhibited OGD (12 h)-induced NO accumulation in the culture medium containing primary cultured astrocytes (Fig. 4D).

**Figure 5:**
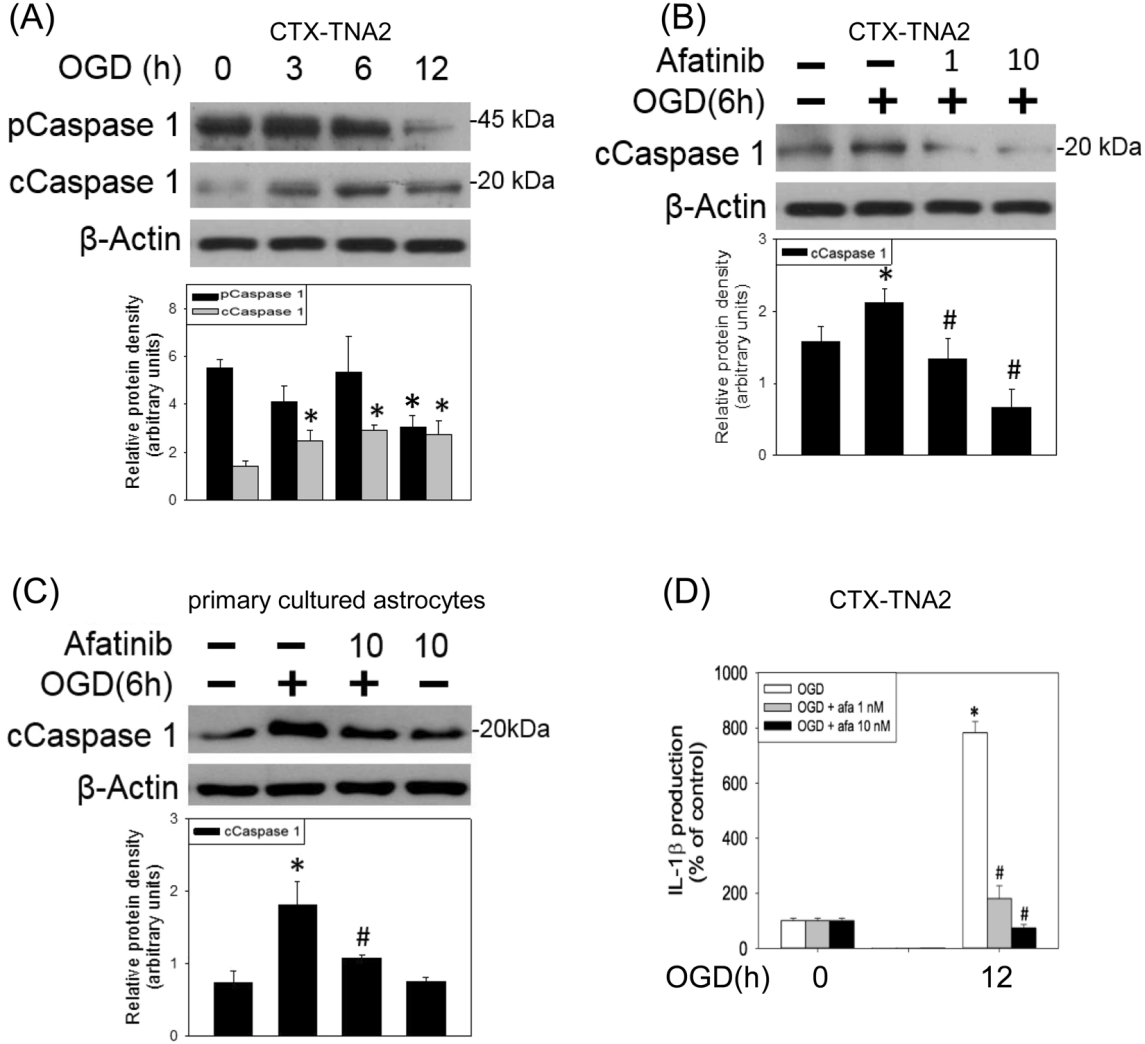
Afatinib prevented OGD-induced inflammasome activation. (**A**) CTX-TNA2 cells were exposed to oxygen-glucose deprivation (OGD) for various durations (3, 6, 12 h). Western blot assay was employed to measure procaspase 1, caspase 1 and β-Actin. Each lane contained 40 μg protein for all experiments. Graphs show statistical results of pCaspase 1 and cCaspase 1 from relative optical density of bands on the blots. Values are the mean ± S.E.M. (n = 3/group). *P < 0.05 in the OGD group compared with the control by t-test. (**B**) CTX-TNA2 cells and (**C**) primary cultured astrocytes were exposed to 6-h OGD. Afatinib (1, 10 nM) was included in the culture medium concomitantly with OGD exposure. Graphs show statistical results of pCaspase 1 and cCaspase 1 from relative optical density of bands on the blots. Values are the mean ± S.E.M. (n = 3/group). (**D**) CTX-TNA2 cells were exposed to OGD for 12 h. Afatinib (1, 10 nM) was included in the culture medium concomitantly with OGD exposure. The levels of IL-1β were measured using ELISA assay. Values are the mean ± S.E.M. (n = 3/group). *P < 0.05 in the OGD group compared with the control, ^#^P < 0.05 in OGD plus afatinib compared with OGD alone by Kruskal-Wallis test and followed by Mann-Whitney U test as post-hoc method.

To study the effect of afatinib on inflammasome formation, both active caspase 1 (an inflammasome specifc caspase) and mature interleukin (IL-1β) formation (a product of caspase 1) were measured using Western blot assay and enzyme-linked immunosorbent assay (ELISA), respectively. OGD reduced procaspase-1 and increased active caspase 1 levels in the CTX-TNA2 cells (Fig. 5A). Concomitant incubation of afatinib reduced OGD (6-h)-induced elevation in active caspase 1 in both CTX-TNA2 cells (Fig. 5B) and primary cultured astrocytes (Fig. 5C). Furthermore, IL-1β in the culture medium of CTX-TNA2 cells was elevated after 12-h OGD; afatinib reduced OGD-indcued elevation in IL-1β in the cultured medium (Fig. 5D). These data suggest that afatinib is capable of inhibiting OGD-induced inflammasome activation in astrocytes.

## Discussion

In the present study, the novel finding is that afatinib is capable of attenuating OGD-induced neuroinflammation in several aspects. First, afatinib blocked OGD-induced EGFR activation and its downstream signalings. Furthermore, afatinib significantly attenuated OGD-induced GFAP expression, immunofluorescent intensities of GFAP and migratory ability of astrocytes. At the same time, afatinib suppressed OGD-induced PCNA immunoreactivities. Moreover, afatinib reduced OGD-elevated COX-II and iNOS expression as well as NO formation. In addition, afatinib inhibited OGD-induced caspase 1 activation and mature IL-1β production. These data suggest that afatinib may exert its anti-inflammatory action by inhibiting OGD-induced EGFR activation, astrocyte activation and proliferation as well as inflammasome activation. Therefore, EGFR-TKIs, such as afatinib may possess an anti-inflammatory activity against neuroinflammation in the CNS neurodegenerative diseases.

Three generations of EGFR-TKIs has been developed for treating non-small lung cancer cells with mutated EGFR^22^. Compared with osimertinib, a third-generation EGFR-TKI which inhibits mutant EGFR but spares wild-type EGFR^23^, afatinib, a second-generation EGFR-TKI, is capable of inhibiting wild-type EGFR^18^. A previous study have reported that afatinib was cytotoxic to murine Ba/F3 cells harboring wild type EGFR with an IC_50_ of 31 nM^24^. We showed that pharmacologically relevant concentrations of afatinib (1 to 100 nM) did not affect the survival of CTX-TNA2 cells and primary cultured astrocytes, indicating no significant neurotoxic effect of afatinib (1 to 100 nM) on astrocytes. In the present study, afatinib at the concentration of 10 nM was chosen to investigate its anti-inflammatory activity because afatinib (10 nM) showed significant inhibition of OGD-induced EGFR activation as well as AKT and ERK phosphorylation in the treated astrocytes. The mechanisms of brain cell responses, including astrocytes, to OGD have been detailed^25^. In the present study, we employed OGD to mimic the involvement of EGFR in brain ischemia *in vivo*^13^, since this treatment reportedly activated EGFR in cultured astrocytes^13,26^. Consistently, our data showed EGFR activation in OGD-treated CTX-TNA2 cells and primary cultured astrocytes. Furthermore, OGD reportedly activates JAK-STAT3^27^, PI3K-AKT and raf-ras-MAPK-ERK pathways in the cultured astrocytes^6^ as well as other known mechanisms in *in vivo* ischemia. To support this notion, we demonstrated that after 3-h OGD, EGFR activation reached the peak levels and phosphorylation of AKT and ERK had just started. Significant phosphorylation of AKT and ERK was observed after 6-h OGD and peaked at 12-h OGD. Several EGFR inhibitors have been investigated, including AG1478 (an EGFR antagonist) used in the middle cerebral artery occlusion model^27^ and C225 (a human-mouse chimeric protein version of anti-EGFR monoclonal antibody EGFR antibody) in traumatic brain injury model^13^. These EGFR inhibitors effectively attenuated brain ischemia only when they were administered intravenously^27^ or intracerebroventricularly^13^. In contrast, we used afatinib which can be delivered via oral administration and is BBB permeable^19,28^. Our data showed that afatinib significantly inhibited OGD-induced EGFR activation and AKT phosphorylation in both cells. Furthermore, afatinib consistently attenuated OGD-induced ERK phosphorylation in CTX-TNA2 cells but showed no effect on primary cultured astrocytes. The mechanism of this inconsistency is unknown. It is possible that ERK activation in primary cultured astrocytes is less sensitive than CTX-TNA2 cells to EGFR-TKIs^29^. Taken together, these findings suggest that afatinib provides an anti-inflammatory strategy against neuroinflammation in the CNS neurodegenerative diseases.

During the brain ischemia, quiescent astrocytes reportedly become reactive astrocytes by augmented GFAP expression in OGD-treated astrocytes^13^. This phenomenon was reproduced in this study that OGD consistently elevated GFAP expression in CTX-TNA-2 cells using the Western blot assay. Furthermore, our immunostaining data showed OGD-induced elevation in co-localized immunoreactivities of GFAP and EGFR, suggesting a permissive role of EGFR of astrocyte activation^11,12^. Moreover, EGFR has been suggested as one of the dependent factors for glial cell migration^30^. In support of these notions, we found that afatinib is capable of inhibiting OGD-induced astrocyte activation and hypoxia-induced migration. Moreover, activated astrocytes are reportedly responsible for the pathophysiology of reactive astrogliosis which leads to cytokine release and glial scar formation^26^. Again, our data showed that afatinib was capable of suppressing OGD-induced astrocyte proliferation. Accordingly, afatinib-induced inhibition of cell proliferation appears to be a promising strategy against astrogliosis in the brain ischemia.

Astrocytic reactivity is one of the sources responsible for neuroinflammation^21^. To mimic neuroinflammation in brain ischemia, OGD-activated astrocytes indeed increased COX-II and iNOS expression. In the present study, afatinib attenuated OGD-induced elevation in COX-II and iNOS expression and NO accumulation which are known to produce oxidative stress and result in oxidative injury^31^. Furthermore, it is clear that cytokines are involved in neurotoxicity in the ischemic brain^32^, and our study is the first to show that OGD induced inflammasome activation, a multiprotein oligomer which activates caspase 1 to cleave proIL-1β and proIL-18 and produce mature IL-1β and IL-18, key mediators of the inflammatory responses^33^. Accordingly, afatinib may exert its anti-inflammatory action by blocking OGD-induced inflammasome activation.

In conclusion, our study provides the evidence for the first time that afatinib, an orally-available EGFR-TKI, could block OGD-induced EGFR activation and its downstream signaling pathways in astrocytes. Moreover, afatinib was capable of attenuating OGD-induced astrocyte activation and proliferation as well as inflammasome activation. These data support a critical role of EGFR activation in neuroinflammation and suggest that EGFR-TKIs may be used as anti-inflammatory agents in treating CNS neurodegenerative diseases.

## Materials and Methods

### Culture of CTX-TNA2 astrocytes

The CTX TNA2 cell line was established from brain frontal cortical tissue of 1-day old rats as primary cultures of type 1 astrocytes. CTX-TNA2 line was maintained in Dulbecco’s Modified Eagle Medium (DMEM) supplemented with 10% (v/v) fetal bovine serum (FBS), 1% penicillin-streptomycin-amphotericin B (PSA) in an incubator under 5% CO_2_ at 37 °C.

### Primary culture of cortical astrocytes

Primary cultured astrocytes were prepared from brain of Sprague-Dawley rat pups (p 5). The rat cortex was dissociated in DMEM/F12 (a 1:1 mixture of DMEM and Ham’s F-12 Medium). The dissociated cells were re-suspended in DMEM/F12 supplemented with 10% (v/v) FBS, 1% PSA and were incubated in 75 cm2 flask coated with poly-L-lysine in an incubator under 5% CO_2_ at 37 °C. The obtained astrocyte-enriched cultures at 7 days–*in-vitro* contained more than 85% astrocytes. The use of animals has been approved by the Institutional Animal Care and Use Committee of Taipei Veterans General Hospital, Taipei, Taiwan, R.O.C. The methods were carried out in accordance with the approved guidelines. All experiments were performed in the accordance with relevant guidelines and regulation. The approval number is IACUC2016-053.

### Oxygen-glucose deprivation (OGD)

For the OGD study, the DMEM deprived of glucose (GD-DMEM) was incubated in 1% oxygen 94% N_2_/5% CO_2_ in a tri-gas incubator (Astec, Japan) overnight to become OGD-DMEM. Next day, cells were rinsed with GD-DMEM and then incubated in OGD-DMEM for indicated duration.

### Drug treatment

Afatinib was purchased from LC laboratories, MA, U.S.A. Afatinib (10 mM) was prepared in dimethyl sulfoxide as stock solution. The effect of afatinib was investigated by treating afatinib concomitantly with OGD exposure. Ten nM Afatinib was chosen according to the concentration of afatinib which significantly attenuated OGD-induced activation of EGFR and subsequent singalling pathways in treated astrocytes.

### Cell viability assay

A modified sulforhodamine B (SRB) assay was employed to measure cell viability. At the end of experiment, cells in 96-well plates were washed with phosphate-buffered saline (PBS) and trichloroacetic acid (10% TCA in water; Merck) and was then incubated at 4 °C for 1 h. Afterwards, TCA solution was removed and the cells were washed with double distilled H_2_O. Cells were then incubated with SRB solution (0.4% in 1% acetic acid, Merck) for 10 min. Afterwards, SRB solution was removed and the cells were washed with 1% acetic acid. After removing acetic acid, the sample was air-dried and 20 mM unbuffered Tris base was used to dissolve the resulting formazan product. The absorption was measured by an ELISA reader at 540 nm with a reference wavelength of 690 nm.

### Cytotoxicity assay

The cytotoxicity was determined by measuring levels of lactate dehydrogenase (LDH) in the culture supernatant. Cells were seeded on a 24-well plate and treated with acrolein for 24 h. The LDH leaked in the culture medium was assessed by addition of reduced β-Nicotinamide adenine dinucleotide and sodium pyruvate. The LDH activity was determined by measuring the absorbance at 340 nm for 6 min using an ELISA reader (TECAN Sunrise, Männedorf, Schweiz). The LDH activity of cells treated with 0.1% Triton X-100 was used as 100% for normalization.

### Western blot analysis of related proteins

At the end of experiment, cells were treated with a RIPA buffer containing Ethylenediaminetetraacetic acid -Na 1 mM, NaCl 0.5 M, Tris 50 mM, sodium dodecyl sulfate (SDS) 0.05%, phenylmethanesulphonylfluoride 1 mM and Triton X-100 0.5%. The lysates were centrifuged at 4 °C, 16500xg for 0.5 h. The supernatant was stored at −80 °C for further analysis. Protein samples (40 μg/lane) were run on and then transferred onto a nitrocellulose membrane (Bio-Rad, Hercules, CA, USA) at 90 V for 120 min. Primary antibodies used to probe blots included phosphorylated EGFR and total EGFR (1:500; Cell Signaling, Beverly, MA); p-AKT and total AKT (1:500; Cell Signaling); pEKR and total ERK (1:3000; Cell Signaling); GFAP (1:2000; Millipore, Billerica, MA), PCNA (1:2000, Millipore #MAB360, Billerica, MA), iNOS (1:500; BD Transduction Lab, San Diego, CA), COX-II (1:1000; Santa Cruz, CA); procaspase 1 and cleaved caspase 1 (1:500; Millipore, Bedford, MA) at 4 °C overnight. Next day, the primary antibodies on membrane were washed; the membrane was then incubated with horseradish peroxidase-conjugated secondary IgG (1:3000; Millipore Billerica, MA) for 1 h at room temperature. The immunoreaction was visualized using Amersham Enhanced Chemiluminescence (Amersham Pharmacia Biotech, Piscataway, NJ, USA). After this detection, stripping buffer (2-mercaptoethanol 100 mM, SDS 2%) was used to strip the bound primary and secondary antibodies incubating the membrane for 45 min at 50 °C. The stripped membrane was reprobed with β-actin antibody (1:5000; Chemicon, Temecula, CA, USA). The densities of blots were analyzed by Image J software and reported as relative optical density of the specific proteins.

### Immunofluorescent staining assay

At the end of experiment, cells were rinsed in PBS and fixed with 3.5% paraformaldehyde (in PBS) for 10 min, permeabilized with 0.5% Triton X-100 in TBS for 30 min, and treated with 2% bovine serum albumin in TBS for 1 h, at room temperature. Cells were then incubated with EGFR (1:200) and GFAP (1:100) overnight at 4 °C, rinsed three times with 0.01% Triton X-100 in TBS, and incubated for 30 min with secondary antibodies, including Fluorescein isothiocyanate-conjugated rabbit IgG (1:250) and rhodamine-conjugated mouse IgG (1:250) at 37 °C. Afterwards, cells were further stained with 4′,6-diamidino-2-phenylindole and observed under a confocal microscopy (Olympus FV1000).

### Cell migration using wound-healing assay

A wound-healing assay was initiated by a scratch in 35 mm tissue culture plates containing CTX-TNA2 cells in normal DMEM cultured medium. Afterwards, the cells were respectively cultured in DMEM in normoxia and hypoxic (1% O_2_) chambers, respectively for 8 h. Microscopic data of the cells at the identical location were taken after the initiation of wound and 8 h after hypoxic treatment. The migratory ability were analyzed by Image J software and reported as the cells that had migrated in the cell-free area.

### NO production using Griess reaction

At the end of experiment, the culture medium was used for NO-production measurement. The culture medium was mixed with an equal volume of the Griess reagent (1% sulfanilamide, 0.1% N-(1-Naphthyl)ethylenediamine in 2.5% H_2_PO_4_) and incubated for 15 min at room temperature in the dark. Nitrite concentration was determined by measuring the absorbance at 550 nm using an ELISA plate reader (TECAN Sunrise, Männedorf, Schweiz)^34^.

### IL-1β levels using enzyme-linked immunosorbent assay (ELISA)

The IL-1β levels of the culture medium containing CTX-TNA2 cells were determined using a rat IL-1β kit (R & D Systems, #RLB00, Minneapolis, MN) and ELISA reader (Molecular Devices Corp, Silicon Valley, CA) according to the manufacturer’s instructions.

### Statistics

All data are expressed as the mean ± S.E.M. Statistical comparisons were made using t test in time-dependent studies of OGD and using Kruskal-Wallis test and followed by Mann-Whitney U test as post-hoc method in afatinib’s effect. Significance level was set at p < 0.05.
