# Author Correction: Anti-inflammatory effect of afatinib (an EGFR-TKI) on OGD-induced neuroinflammation

**DOI:** 10.1038/s41598-020-80901-1

**Published:** 2021-01-25

**Authors:** Yen-Ju Chen, Chia-Chi Hsu, Young-Ji Shiao, Hsiang-Tsui Wang, Yu-Li Lo, A. M. Y. Lin

**Affiliations:** 1 Institute of Pharmacology, National Yang-Ming University, Taipei, Taiwan; 2 Department of Oncology, National Taiwan University Hospital, Taipei, Taiwan; 3 National Research Institute of Chinese Medicine, Ministry of Health and Welfare, Taipei, Taiwan; 4 Faculty of Pharmacy, National Yang-Ming University, Taipei, Taiwan; 5 Department of Medical Research, Taipei-Veterans General Hospital, Taipei, Taiwan

Correction to: *Scientific Reports* 10.1038/s41598-019-38676-7, published online 21 February 2019

This Article contains errors in Figure 2 and the accompanying Figure legend.

In panel C, in the bar graph showing Relative protein intensity (arbitrary units) for pEGFR, an asterisk (*) is incorrectly shown in the third bar.

In panel D, the representative blot of pAKT is incorrect. Additionally, a representative blot for AKT is incorrectly shown. Instead, a representative blot for β-Actin should be shown. Finally, in the bar graph showing Relative protein intensity (arbitrary units) for pAKT and pERK, a hash sign (#) is incorrectly shown in the fifth and sixth bar.

In panel E, in the bar graph showing Relative protein intensity (arbitrary units) for pEGFR, a hash sign (#) is omitted in the fourth bar.

In panel F, in the bar graph showing Relative protein intensity (arbitrary units) for pEGFR and pERK, a hash sign (#) is omitted in the seventh bar.

Lastly, in the Figure legend,

“Afatinib blocked OGD-induced EGFR activation and downstream signalings. (**A**,**B**) CTX-TNA2 cells were exposed to oxygen-glucose deprivation (OGD) for various durations (3, 6, 12 h). Western blot assay was employed to measure (**A**) the levels of phospho-EGFR (pEGFR) and total-EGFR (EGFR) as well as (**B**) phospho-ERK (pERK), total-ERK (ERK), phospho-AKT (pAKT), total-AKT (AKT) and β-Actin. Each lane contained 40 μg protein for all experiments. Graphs show statistical results of pEGFR and EGFR (**A**) as well as pERK, ERK, pAKT and AKT (**B**) from relative optical density of bands on the blots. *P < 0.05 in the OGD group compared with the control by t-test. (**C**,**D**) CTX-TNA2 cells were exposed to OGD for 12 h. Afatinib (1, 10 nM) was included in the culture medium concomitantly with OGD exposure. (**E**,**F**) Primary cultured astrocytes were exposed to OGD for 12 h. Afatinib (10 nM) was included in the culture medium concomitantly with OGD exposure. Graphs show statistical results of pEGFR and EGFR (**C**,**E**) as well as pERK, ERK, pAKT and AKT (**D**,**F**) from relative optical density of bands on the blots. Values are the mean ± S.E.M. (n = 3/group). *P < 0.05 in the OGD group compared with the control, ^#^P < 0.05 in OGD plus afatinib compared with OGD alone by Kruskal-Wallis test and followed by Mann-Whitney U test as post-hoc method.”

should read:

“Afatinib blocked OGD-induced EGFR activation and downstream signalings. (**A**,**B**) CTX-TNA2 cells were exposed to oxygen-glucose deprivation (OGD) for various durations (3, 6, 12 h). Western blot assay was employed to measure (**A**) the levels of phospho-EGFR (pEGFR) and total-EGFR (EGFR) as well as (**B**) phospho-ERK (pERK), total-ERK (ERK), phospho-AKT (pAKT), total-AKT (AKT) and β-Actin. Each lane contained 40 μg protein for all experiments. Graphs show statistical results of pEGFR (**A**) as well as pERK and pAKT (**B**) from relative optical density of bands on the blots. *P < 0.05 in the OGD group compared with the control by t-test. (**C**,**D**) CTX-TNA2 cells were exposed to OGD for 12 h. Afatinib (1, 10 nM) was included in the culture medium concomitantly with OGD exposure. (**E**,**F**) Primary cultured astrocytes were exposed to OGD for 12 h. Afatinib (10 nM) was included in the culture medium concomitantly with OGD exposure. Graphs show statistical results of pEGFR (**C**,**E**) as well as pERK and pAKT (**D**,**F**) from relative optical density of bands on the blots. Values are the mean ± S.E.M. (n = 3/group). n.a. is not applicable because these experiments were performed only in duplicate (n = 2) and so the comparison was not performed. *P < 0.05 in the OGD group compared with the control, ^#^P < 0.05 in OGD plus afatinib compared with OGD alone by Kruskal-Wallis test and followed by Mann-Whitney U test as post-hoc method.”

The correct Figure 2 appears below as Figure 1.

**Figure 1:**
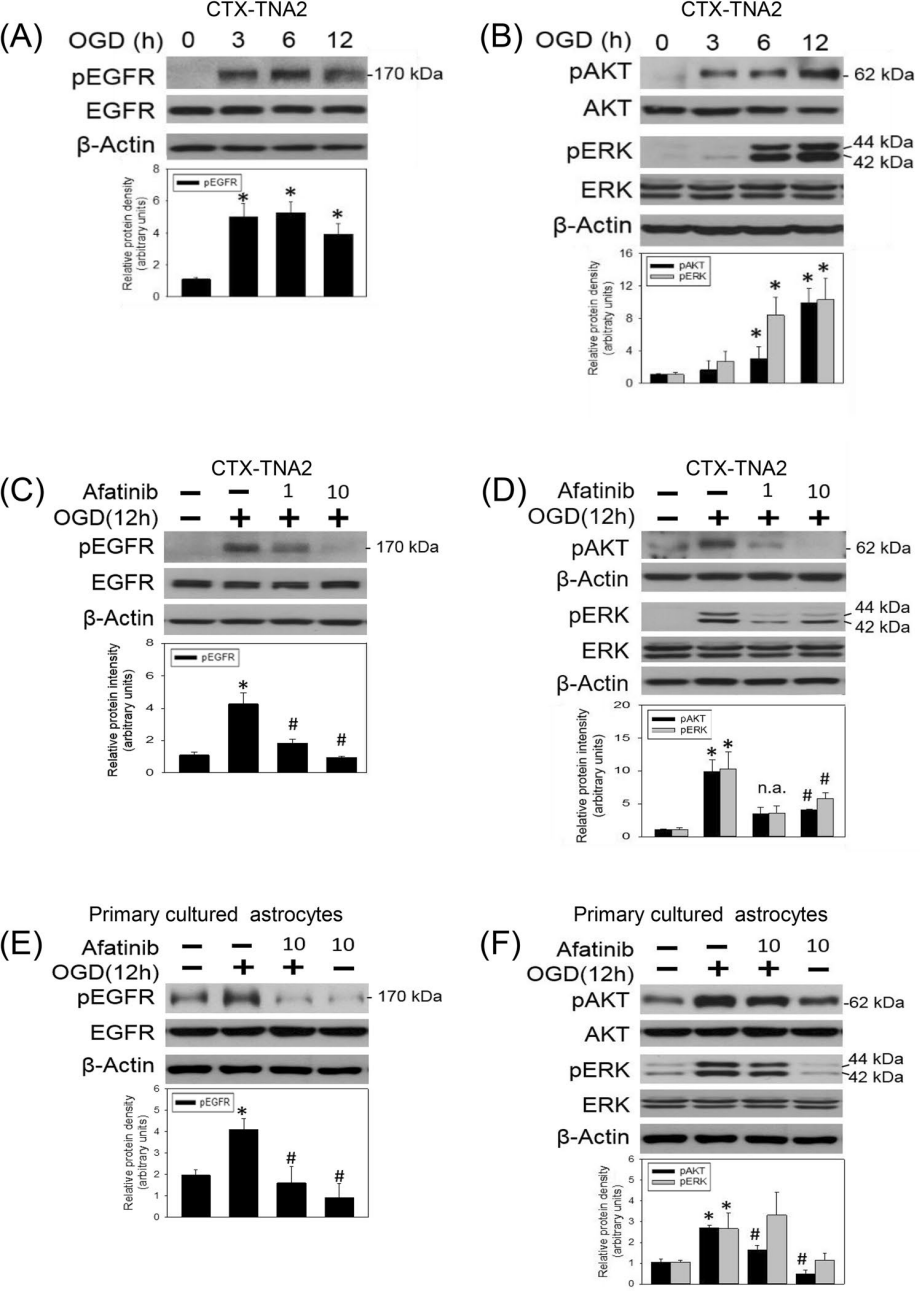
A corrected version of the original Figure 2. Afatinib blocked OGD-induced EGFR activation and downstream signalings. (**A**,**B**) CTX-TNA2 cells were exposed to oxygen-glucose deprivation (OGD) for various durations (3, 6, 12 h). Western blot assay was employed to measure (**A**) the levels of phospho-EGFR (pEGFR) and total-EGFR (EGFR) as well as (**B**) phospho-ERK (pERK), total-ERK (ERK), phospho-AKT (pAKT), total-AKT (AKT) and β-Actin. Each lane contained 40 μg protein for all experiments. Graphs show statistical results of pEGFR (**A**) as well as pERK and pAKT (**B**) from relative optical density of bands on the blots. *P < 0.05 in the OGD group compared with the control by t-test. (**C**,**D**) CTX-TNA2 cells were exposed to OGD for 12 h. Afatinib (1, 10 nM) was included in the culture medium concomitantly with OGD exposure. (**E**,**F**) Primary cultured astrocytes were exposed to OGD for 12 h. Afatinib (10 nM) was included in the culture medium concomitantly with OGD exposure. Graphs show statistical results of pEGFR (**C**,**E**) as well as pERK and pAKT (**D**,**F**) from relative optical density of bands on the blots. Values are the mean ± S.E.M. (n = 3/group). n.a. is not applicable because these experiments were performed only in duplicate (n = 2) and so the comparison was not performed. *P < 0.05 in the OGD group compared with the control, ^#^P < 0.05 in OGD plus afatinib compared with OGD alone by Kruskal-Wallis test and followed by Mann-Whitney U test as post-hoc method.

